# An evaluation of the sensitivity of acute flaccid paralysis surveillance for poliovirus infection in Australia

**DOI:** 10.1186/1471-2334-9-162

**Published:** 2009-09-30

**Authors:** Rochelle E Watkins, P Anthony J Martin, Heath Kelly, Ben Madin, Charles Watson

**Affiliations:** 1 Australian Biosecurity CRC for Emerging Infectious Disease, Faculty of Health Sciences, Curtin University of Technology, Perth, Australia; 2 Department of Agriculture and Food, Bunbury, Australia; 3 Victorian Infectious Diseases Reference Laboratory, North Melbourne, Australia; 4 AusVet Animal Health Services, Broome, Australia

## Abstract

**Background:**

World Health Organization (WHO) targets for acute flaccid paralysis (AFP) surveillance, including the notification of a minimum rate of AFP among children, are used to assess the adequacy of AFP surveillance for the detection of poliovirus infection. Sensitive surveillance for poliovirus infection in both developed and developing countries is essential to support global disease eradication efforts. We applied recently developed methods for the quantitative evaluation of disease surveillance systems to evaluate the sensitivity of AFP surveillance for poliovirus infection in Australia.

**Methods:**

A scenario tree model which accounted for administrative region, age, population immunity, the likelihood of AFP, and the probability of notification and stool sampling was used to assess the sensitivity of AFP surveillance for wild poliovirus infection among children aged less than 15 years in Australia. The analysis was based on historical surveillance data collected between 2000 and 2005. We used a surveillance time period of one month, and evaluated the ability of the surveillance system to detect poliovirus infection at a prevalence of 1 case per 100 000 persons and 1 case per million persons.

**Results:**

There was considerable variation in the sensitivity of AFP surveillance for poliovirus infection among Australian States and Territories. The estimated median sensitivity of AFP surveillance in Australia among children aged less than 15 years was 8.2% per month at a prevalence of 1 case per 100,000 population, and 0.9% per month at a prevalence of 1 case per million population. The probability that Australia is free from poliovirus infection given negative surveillance findings following 5 years of continuous surveillance was 96.9% at a prevalence of 1 case per 100,000 persons and 56.5% at a prevalence of 1 case per million persons.

**Conclusion:**

Given the ongoing risk of poliovirus importation prior to global eradication, long term surveillance is required to provide a high degree of confidence in freedom from poliovirus infection in Australia, particularly if a low prevalence of infection is assumed. Adherence to the WHO surveillance targets would considerably improve the sensitivity of surveillance for poliovirus infection in Australia.

## Background

The Global Polio Eradication Initiative commenced in 1988 following the adoption of a resolution to eradicate poliomyelitis (polio) at the World Health Assembly [1]. The worldwide polio eradication campaign has been successful in achieving a 99% reduction in the global incidence of polio since 1988 [2]. The number of countries that have not yet interrupted indigenous transmission of wild poliovirus was reduced to 4 in 2006 [3], although the final stages of eradication are proving challenging [4].

Since 2003, at least 22 countries that were previously polio-free have reported re-infection [5]. Three large outbreaks following importation into the previously polio-free countries of Yemen, Indonesia and Somalia were largely responsible for the increase in reported cases of polio during 2005, and this was the first year in which more cases were reported from re-infected countries than from endemic countries [6]. These outbreaks emphasise the importance of effective vaccination, disease surveillance and response systems to polio eradication efforts, irrespective of the local disease incidence [7].

Poliovirus is an enterovirus which has no extrahuman reservoir [8]. In developing countries, polio primarily affects infants and children less than 5 years of age. Infection is spread most commonly by the faecal-oral route, particularly in the presence of poor hygiene and sanitation. Oral-oral respiratory transmission is thought to be more common in industrialised countries [9].

In most people poliovirus infection is subclinical. A small proportion of cases experience viral replication in the central nervous system which may lead to permanent neuronal destruction and paralysis [9]. As a result, poliovirus infection is most commonly recognised by the onset of acute flaccid paralysis (AFP). Acute flaccid paralysis is estimated to occur in between 0.1% and 2% of poliovirus infections, with residual paralysis occurring in between 0.1% and 1% of infections [9]. The high proportion of subclinical cases contributes to difficulties in disease eradication [10]. Laboratory-based surveillance for poliovirus infection among reported cases of AFP is used to monitor and control poliovirus infection, as there are no criteria that permit the identification of polio by clinical signs and symptoms alone.

In October 2000, the World Health Organisation (WHO) certified the Western Pacific Region, which includes Australia, as polio-free [11]. To be certified as polio-free, countries must have an absence of wild poliovirus for 3 years in the presence of adequate AFP surveillance in children under the age of 15 years, have a national committee to validate and submit the surveillance documentation, and have mechanisms to detect and respond to the introduction of wild poliovirus [12]. Prior to the 2007 importation of wild poliovirus into Australia via a traveller returning from Pakistan [13], the last recorded importation of wild poliovirus in Australia occurred in 1977 [14].

The Australian Paediatric Surveillance Unit commenced AFP surveillance in children aged less than 15 years in 1995 [15]. In order to maximise the ability of surveilance to rapidly detect imported cases, World Health Organization guidelines require all cases of AFP in children aged less than 15 years be notified and investigated as prospective polio cases, including the collection of 2 stool samples 24 hours apart and within 14 days of the onset of paralysis [15]. The requirement to investigate all cases of AFP is considered critical to support the sensitivity of polio surveillance. Notification of AFP and stool investigation allow the detection of poliovirus transmission through the identification of both classical and atypical cases, as well as providing a basis for assessment of the quality of surveillance [12].

The established performance indicators for AFP surveillance require that reporting should be complete, timely, and represent the geography and demography of the country; that at least one case of non-polio AFP should be detected annually per 100,000 population aged less than 15 years; that full clinical and virological investigations should be completed for all AFP cases, with at least 80% having adequate faecal samples collected for analysis at an accredited laboratory; and that follow-up examination for residual paralysis should occur in at least 80% of cases [12].

Effective AFP surveillance in countries which have been declared polio-free is essential to support polio eradication through the detection of imported and vaccine-associated polio cases, and AFP surveillance is required for countries to retain their polio-free status [16]. Historically, Australia has been unable to maintain sufficiently sensitive AFP surveillance [17,18] as the reported incidence of AFP in Australia has often been below the expected rate. Formal quantitative evaluation of AFP surveillance is required to ensure that surveillance is adequate to detect poliovirus infection at an appropriate prevalence.

This analysis aimed to use existing data and scenario tree modelling to provide an improved understanding of the sensitivity of AFP surveillance among children aged less than 15 years in Australia. Scenario tree modelling allows explicit quantification of the sensitivity of AFP surveillance, and examines the impact of multiple factors on surveillance performance. Comprehensive assessment of surveillance system performance can facilitate the efficient allocation of resources for disease surveillance and control.

## Methods

We used a stochastic scenario tree model [19] to evaluate the sensitivity of AFP surveillance for wild poliovirus infection among children aged less than 15 years in Australia, and estimate the probability of Australia of being free from wild poliovirus infection on the basis of this surveillance. The model has two main assumptions: that all surveillance results in the period under analysis are negative, and that the surveillance system does not produce false positive results [19]. The second assumption of perfect specificity implies that adequate information is available for each eligible case of AFP identified to resolve potential false positive results and allow a definitive diagnosis to be made.

The assumption of perfect specificity is supported by WHO surveillance standards [12] which require a minimum rate of AFP case notification and stool sampling, the timely and complete collection of clinical information, follow-up assessment for a minimum of 80% of cases, and the use of accredited laboratories and an expert review committee. Stool testing is considered the gold standard for the exclusion of polio in the presence of AFP [15].

Scenario tree modelling requires the surveillance outcome to be dichotomous. Within the existing AFP surveillance system three outcomes are possible for notified AFP cases: confirmed poliomyelitis due to poliovirus, non-poliomyelitis AFP, and poliomyelitis compatible. The latter classification is used in cases where the surveillance system failed to collect adequate specimens within the recommended time to allow definitive case classification. Although potentially poliomyelitis compatible cases were reported during the study period (2000-2005), no cases were classified as poliomyelitis compatible during the study period, providing data that are consistent with a dichotomous outcome model.

**Figure 1:**
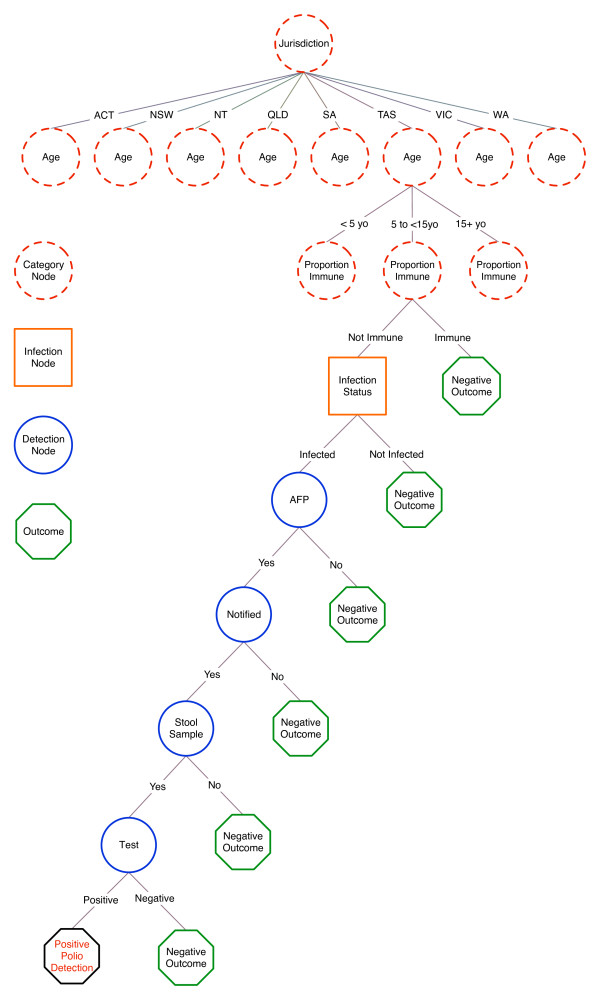
**Scenario tree structure for acute flaccid paralysis surveillance in Australia**.

A scenario tree model was constructed to describe the clinical diagnostic system used for AFP surveillance among children aged less than 15 years in Australia, with the surveillance unit being individuals. Variables that describe the structure of the population and influence the probability of disease or the probability of detecting disease, including factors which may bias sampling, were incorporated in the scenario tree as nodes. The scenario tree structure is summarised in Figure 1. The clinical decision-making process and the Australian Polio Expert Committee surveillance data review process are not represented in the scenario tree as they are not assumed to be associated with any decrease in the sensitivity of the surveillance system. Justification for node selection and the source of parameter estimates is detailed in the following section, and the parameters used are summarised in Table 1. This research was approved by the Human Research Ethics Committee of Curtin University of Technology.

**Table 1:** Parameter estimates used in the scenario tree analysis of acute flaccid paralysis surveillance in Australia

Node	Category	Probability or proportion
Jurisdiction	ACT	0.016
	NSW	0.330
	NT	0.010
	QLD	0.197
	SA	0.076
	TAS	0.024
	VIC	0.248
	WA	0.099
Age	<5 years	
	ACT	0.062
	NSW	0.064
	NT	0.082
	QLD	0.066
	SA	0.058
	TAS	0.060
	VIC	0.062
	WA	0.064
	5 to <15 years	
	ACT	0.129
	NSW	0.134
	NT	0.165
	QLD	0.141
	SA	0.128
	TAS	0.137
	VIC	0.131
	WA	0.139
Proportion immune	Immune	
	<5 years	(0.85,0.91,0.95)^†^
	5 to <15 years	(0.81,0.87,0.91)^†^
	15+ years	(0.75,0.80,0.85)^†^
Infection status	Infected	0.00001
Acute Flaccid Paralysis	Yes	
	<5 years	(0.001,0.005,0.01)^†^
	5 to <15 years	(0.005,0.01,0.02)^†^
	15+ years	(0.005,0.01,0.02)^†^
Notified	Yes	
	ACT	Beta(1.3,61931.7)^‡^
	NSW	Beta(18.5,1298900.5)
	NT	Beta(2.2,47604.8)^‡^
	QLD	Beta(11.3,806522.7)^‡^
	SA	Beta(2.7,280823.3)^‡^
	TAS	Beta(1.7,94022.3)^‡^
	VIC	Beta(6.2,950388.8)^‡^
	WA	Beta(2.7,396433.3)^‡^
Stool sample	Yes	
	ACT	Beta(2,2)
	NSW	Beta(23,84)
	NT	Beta(1,8)
	QLD	Beta(29,35)
	SA	Beta(3,9)
	TAS	Beta(2,4)
	VIC	Beta(10,23)
	WA	Beta(4,8)
Test	Positive	(0.95,0.97,0.99)^†^

^†^Beta-PERT (Program Evaluation and Review Technique) distribution (lower, most likely, upper)

^‡^Calculated as the ratio of this beta distribution divided by 10^-5^, with the result constrained to have an upper limit of 1.

### Nodes and node probabilities

#### Jurisdiction

State and Territory governments are principally responsible for providing clinical health services to the resident population within each of these administrative subdivisions. A category node was incorporated into the model to reflect the underlying structure of health service delivery and reporting, and allow an assessment of surveillance sensitivity by jurisdiction. Branch proportions describe the proportion of the Australian population residing in each State or Territory based on data from the 2006 Census [20].

#### Age

Age was incorporated into the model to reflect the age-specific targeting of AFP surveillance. Age is also a risk factor for infection [9], with the incidence of poliovirus infection decreasing with age in the absence of immunisation. Infection later in life is also associated with increased disease severity [21]. Three age categories were incorporated in the scenario tree model. Branch proportions describe the proportion of the population aged less than 5 years, 5 to less than 15 years, and 15 years or more, based on data from the 2006 Census [20].

#### Proportion immune

A risk category node was incorporated in the model to represent the population prevalence of immunity to poliovirus infection. Live attenuated (oral) polio vaccine was the predominant vaccine used during the study period; it was removed from the Australian immunisation schedule in November 2005 [22]. Two indicators of the population prevalence of immunity were available: estimates from a national serosurvey based on sera collected between 1996 and 1999 [23], and data from the Australian Childhood Immunisation Register (ACIR) [24] which are considered to give a minimum estimate of vaccination coverage due to delayed notification and vaccination [25,26]. Serosurvey data are considered to provide a better indicator of the proportion of the population who are susceptible to poliovirus infection, as immune status is also influenced by naturally acquired immunity and waning immunity [27]. Serosurvey estimates also eliminate potential biases associated with jurisdiction-specific differences in immunisation notifications [28].

National serosurvey point estimates and 95% confidence intervals for immunity to wild type 1 poliovirus infection [23] were used to parameterise Beta-PERT distributions (Table 1) as wild type 1 poliovirus is the virus most commonly associated with importations and outbreaks [29]. Age-specific serosurvey estimates were weighted by population proportions [20] to provide estimates for the age categories used in this analysis. Due to a lack of evidence on the strength of the protective effect associated with being sero-positive [23], we estimated that the risk of infection among sero-positive individuals compared with sero-negative individuals as varying between 1/500 and 1/1500 with a mean of 1/1000.

Although the prevalence of immunity to poliovirus infection is an important factor influencing the likelihood of infection, this variable does not affect the estimated sensitivity of surveillance in the current scenario tree model, as there is no differential surveillance of cases according to sero-status. That is, in this model the detection node parameters are identical for both the sero-positive and sero-negative individuals, and as such, parameter estimates based on serosurvey data and ACIR data produce equivalent results. However, the prevalence of immunity is included in this analysis for completeness, and will be an essential component of models that incorporate targeted surveillance strategies based on susceptibility to infection.

#### Infection status

As the sensitivity of a surveillance system depends on the prevalence of infection in the population, evaluation needs to be performed with reference to a selected population prevalence of infection, or 'design prevalence' [30], which is specified at the infection status node of the scenario tree. The sensitivity of AFP surveillance was evaluated based on infection being present at or above design prevalences of 10^-5 ^(approximately 199 infections expected nationally) and 10^-6 ^(approximately 20 infections expected nationally). A population prevalence of infection of 10^-6 ^may approach a level where the odds of the virus persisting in the population are low [31].

The infection status node also incorporates the effect of age on the risk of infection. Developing countries with endemic poliovirus transmission typically observe most paralytic poliomyelitis cases in children below 5 years of age [9,32]. Children are thought to play a dominant role in the transmission of polioviruses within populations [27], although the effect of age alone on the prevalence and severity of poliomyelitis is difficult to specify with confidence [33]. Poor sanitation and hygiene is thought to contribute to the age profile of cases in developing countries [34]. In developed countries, population immunity, including historical deficiencies in vaccination coverage, is likely to be the most important determinant of the age distribution of poliomyelitis cases [35,36].

Two different estimates were used to describe the age-related risk of poliovirus infection due to uncertainty associated with the effect of age. For the first, no difference in the risk of infection by age was specified. For the second, parameters were based on published estimates of the age distribution of poliomyelitis cases in developed countries in the absence of immunisation [9]. The risk of infection for persons aged less than 5 years was 3 times greater than for persons aged 15 years or more, and the risk of infection for persons aged 5 to less than 15 years was 2 times greater than for persons aged 15 years or more.

#### Acute Flaccid Paralysis

The probability of individuals with poliovirus infection having AFP is an important determinant of case detection rates, and is commonly estimated to vary between 0.001 and 0.01 or 0.02 [21,37,38], with a mean around 0.005 [38,39]. Difficulties in detecting and defining non-paralytic poliovirus infections, and accounting for the effects of prior immunity add to the uncertainty in age-specific rates of AFP [33].

Data from epidemics among largely naive populations suggest that young children are likely to have mild infections, and that adults are more likely to have severe paralytic infections [33]. Another review [21] also suggests that there is an increase in the ratio of paralytic cases to infections with increasing age, with an approximate two-fold increase in risk of paralytic rates among older children or young adults compared with younger children. Based on these data [21], we specified the rate of AFP among persons aged 5 years or more to be twice the rate among individuals aged less than 5 years.

#### Notified

Paediatric cases of AFP are considered to come to the attention of the health care system in developed countries due to the severity of the condition [40]. For each Australian jurisdiction, the probability of notification of a case of AFP was based on a comparison of the average probability of notification of AFP cases between 2000 and 2005 per 100,000 to the recommended AFP case notification rate of 1 per 100,000 children aged less than 15 years [12], using data provided by the Australian National Poliovirus Reference Laboratory. A beta distribution was used to model the overall probability of notification between 2000 and 2005 for each jurisdiction, and the ratio of this probability per 100,000 population to the expected notification rate (constrained to have an upper limit of 1.0) was used in the scenario tree. There is little evidence to suggest that the true AFP notification rate in Australia is lower than that designated by the WHO [41,42].

#### Stool sample

The probability of adequate clinical samples being obtained for notified cases was modelled using a beta distribution based on the proportion of notified cases for each jurisdiction between 2000 and 2005 that had two stool samples submitted for analysis within the recommended time frame, and the number of AFP cases notified by each jurisdiction over the same period.

#### Test

The final detection node represents the diagnostic sensitivity of stool testing for the presence of poliovirus. A published estimate of the sensitivity of laboratory testing for poliovirus based on nine years of AFP surveillance data of 97% [43] was used as the basis for the sensitivity estimate used in the model, allowing for uncertainty. Comprehensive follow-up testing of positive test results ensures perfect specificity.

### Calculation of sensitivity

The sensitivity of the surveillance system was evaluated per month due to the reasonably short incubation period of polio, with cases generally occurring 1-3 weeks following exposure [9]. The unit sensitivity of AFP surveillance (the probability that an individual will yield a positive surveillance finding, given that the population is infected at the design prevalence) is calculated by summing the probabilities for all positive outcomes of the scenario tree [19]. The probability of each positive outcome of the scenario tree is calculated by multiplying all node probabilities, proportions and risks associated with that outcome.

To calculate the system sensitivity of the surveillance system (SSe) the unit sensitivity is raised to the power of the number of units processed in the surveillance system (the Australian population), using Equation 1. The system sensitivity for each jurisdiction was computed similarly, based on the population proportion in each jurisdiction.(1)

To estimate the probability of freedom from poliovirus infection, a prior estimate of the level of certainty of Australia being free from wild poliovirus infection is required for the initial surveillance month. Although historical surveillance data suggest that the probability of Australia being free from poliovirus infection is high, an imported case was detected in 2007 [13]. The failure of poliovirus to establish in Australia given importation can be attributed to high levels of population immunity; however, immunity is not uniformly distributed and outbreaks could still occur. A neutral prior probability of 0.5 was used to provide a conservative estimate of the prior certainty of freedom from poliovirus infection following a known importation.

To estimate the probability of freedom from poliovirus infection for subsequent months of surveillance, the value of surveillance data must be discounted, as infection may be imported, or may have been present in the population during the previous surveillance period but remained undetected. In the 30-year period between 1978 and 2007, only 1 imported case of wild poliovirus infection was detected. Based on historical data, the probability of poliovirus introduction into Australia is therefore 1/360 per month (assuming that all imported cases are detected), and the probability of establishment of wild poliovirus infection in Australia at the minimum design prevalence of 10^-6 ^is 0/360 (assuming all outbreaks have been detected). This estimate can be modelled as a Beta(1,361) distribution based on the Bayesian estimate of a population proportion derived from the observation of s successes out of n samples being Beta(s+1, n-s+1) [44].

A more conservative estimate of the rate of importation of wild poliovirus infection into Australia would account for the likelihood of asymptomatic infection and disease establishment. Given the likelihood of asymptomatic infection decreases with age, the probability of wild poliovirus introduction in travellers is likely to be approximately 100 times the detected importation rate, or 1/3.6 per month. Based on recent work indicating a very low risk of wild poliovirus outbreaks in high income countries [39], we conservatively estimated the risk of establishment of poliovirus infection in Australia given introduction to be 0.001. A review of wild poliovirus importation into 21 previously polio-free countries [29] also suggests that uniformly high vaccination coverage levels in Australia are strongly protective against the establishment of introduced wild poliovirus infection. The probability of introduction and establishment, accounting for the likely proportion of asymptomatic infection, was estimated to be 1/3600 (1/3.6*1/1000), and was modelled as a Beta(2,3600) distribution.

### Simulation parameters

The model was implemented using Microsoft Excel 2003. The @RISK add-in for Excel version 4.5.2 (Palisade Corporation) was used to estimate the sensitivity of the surveillance system and perform stochastic simulations by sampling from the specified distributions in the model. The simulation used a fixed random number seed of 1, and 10,000 iterations were performed using latin hypercube sampling. Results are summarised using the median and range (5^th^-95^th ^percentiles) of the outcome variable distributions.

### Comparison with WHO criteria

The sensitivity of AFP surveillance based on historical data was compared with the sensitivity of surveillance implied by the WHO guidelines (assuming all States and Territories notify cases of AFP at a rate of 1 per 100,000 or higher and have an 80% probability of submitting two stool samples) as a sensitivity ratio [19]. All other node probabilities and relative risks remained consistent between the two sensitivity estimates.

## Results

### Surveillance system sensitivity

The estimated median monthly sensitivity of AFP surveillance for poliovirus infection in Australia among children aged less than 15 years, assuming no difference in the risk of infection with age, was 8.2% (5^th^-95^th ^percentiles 5.3-12.1%, mean 8.4%) for a design prevalence of 10^-5^, and 0.9% (5^th^-95^th ^percentiles 0.5-1.3%, mean 0.9%) for a design prevalence of 10^-6 ^respectively. These results represent the probability of observing one or more positive test results for poliovirus in a child with AFP if the population is infected at the design prevalence.

If the risk of infection was greater among younger children, the estimated median monthly sensitivity of AFP surveillance almost doubled to 13.8% (5^th^-95^th ^percentiles 9.1-19.7%, mean 14.0%) for a design prevalence of 10^-5^, and 1.5% (5^th^-95^th ^percentiles 1.0-2.2%, mean 1.5%) for a design prevalence of 10^-6 ^respectively.

The probability of freedom from poliovirus infection in Australia given consistent negative surveillance findings is summarised in Table 2. Results assume a conservative initial prior probability of infection (0.5), use the highest estimate of the probability of disease introduction (Beta(1,361)), and allow for different assumptions about the desired design prevalence for surveillance and the distribution of infection with age.

**Table 2:** Probability of freedom from poliovirus infection in Australia given continuous negative surveillance findings

	Probability of freedom (%)^‡^			
**Design prevalence**	**10^-5^**		**10^-6^**	

**Time period**	**No age effect**	**Age effect**	**No age effect**	**Age effect**

6 months	61.9 (57.3-68.7)	70.2 (63.2-78.3)	50.1 (49.2-51.6)	51.6 (50.0-52.9)
1 year	72.2 (63.8-81.2)	84.2 (74.1-92.4)	51.4 (48.0-53.2)	53.2 (49.7-55.7)
2 years	86.2 (74.6-94.3)	95.6 (87.5-98.9)	52.8 (45.9-56.3)	56.2 (49.0-61.2)
3 years	92.8 (81.8-98.1)	98.0 (92.5-99.7)	54.0 (43.9-59.3)	59.0 (48.4-66.3)
4 years	95.6 (85.9-99.1)	98.6 (93.9-99.8)	55.3 (42.1-62.2)	61.8 (47.8-71.0)
5 years	96.9 (88.0-99.5)	98.7 (94.2-99.9)	56.5 (40.4-65.0)	64.3 (47.3-75.1)
10 years	97.8 (89.9-99.8)	98.8 (94.4-99.9)	61.9 (33.6-76.9)	74.5 (45.3-89.0)
20 years	97.8 (90.0-99.9)	98.8 (94.4-99.9)	69.3 (25.1-90.0)	83.8 (43.1-97.0)

^‡^Median (5^th^-95^th ^percentile), probability of introduction Beta(1,361)

The probability of Australia being free from poliovirus infection after 5 years of continuous negative surveillance results, assuming no difference in the risk of infection with age, was 96.9% for a design prevalence of 10^-5^, and 56.5% for a design prevalence of 10^-6^. If the risk of infection was greater among younger children, the probability of Australia being free from poliovirus infection after 5 years was 98.7% for a design prevalence of 10^-5^, and 64.3% for a design prevalence of 10^-6 ^(Table 2). Surveillance for poliovirus infection at a design prevalence of 10^-6 ^is only able to support a high probability of freedom from infection over long periods of surveillance, and the 5^th ^and 95^th ^percentile estimates indicate a high degree of variability in the probability of freedom over long surveillance periods.

Given a lower probability of disease introduction (Beta(2,3600)), the probability of Australia being free from poliovirus infection after 5 years, assuming no difference in the risk of infection with age, was 98.8% (5^th^-95^th ^percentiles 95.3-99.7%) for a design prevalence of 10^-5^, and 60.9% (5^th^-95^th ^percentiles 56.0-66.9%) for a design prevalence of 10^-6^. If the risk of infection was greater among younger children, the probability of Australia being free from poliovirus infection after 5 years was 99.7% (5^th^-95^th ^percentiles 98.9-99.9%) for a design prevalence of 10^-5^, and 69.2% (5^th^-95^th ^percentiles 62.0-77.5%) for a design prevalence of 10^-6^.

### Comparison with WHO recommended surveillance

Historical data describing the rate of notification and stool sampling among AFP cases in each jurisdiction between 2000 and 2005 are presented in Table 3. All 95% confidence intervals for AFP notification rates include 1 case per 100,000 children aged less than 15 years apart from estimates for Victoria and Western Australia. The exact binomial confidence interval is known to be conservative for rare events [45]; however, findings were not appreciably different when the score method was used (data not shown). Similarly, 95% confidence intervals for the probability of stool sampling given notification do not include the WHO target rate for all jurisdictions apart from the Australian Capital Territory and Tasmania, which both have wide confidence intervals due to the small number of cases observed.

**Table 3:** State and territory surveillance system sensitivity (2000-2005) at a design prevalence of 10^-5^

Jurisdiction	Notification rate per 100,000/year*	Probability of stool sampling**	Median sensitivity (%)^† ^(5^th^-95^th ^percentiles)	Sensitivity Ratio^‡^
ACT	0.54 (0.07-2.78)	0.50 (0.01-0.99)	0.21 (0.03-0.49)	0.50
NSW	1.35 (1.10-1.63)	0.21 (0.01-0.30)	2.31 (1.39-3.61)	0.27
NT	2.45 (0.99-5.05)	0.00 (0.00-0.41)	0.03 (0.002-0.13)	0.09
QLD	1.28 (0.98-1.64)	0.45 (0.32-0.58)	2.99 (1.87-4.54)	0.55
SA	0.59 (0.28-1.09)	0.20 (0.03-0.56)	0.40 (0.08-1.05)	0.21
TAS	0.71 (0.19-1.82)	0.25 (0.01-0.81)	0.20 (0.03-0.54)	0.31
VIC	0.54 (0.37-0.77)	0.29 (0.14-0.48)	1.44 (0.55-3.09)	0.23
WA	0.42 (0.20-0.77)	0.30 (0.07-0.65)	0.60 (0.13-1.62)	0.22
Australia	0.98 (0.86-1.11)	0.29 (0.23-0.35)	8.22 (5.32-12.06)	0.35

*Rate per 100,000 persons aged < 15 years per year, (lower 95% binomial exact CI: upper 95% binomial exact CI).

**Probability (lower 95% binomial exact CI: upper 95% binomial exact CI).

^†^Model assumes no influence of age on the likelihood of infection.

^‡^Ratio of the jurisdiction sensitivity and the WHO recommended jurisdiction sensitivity.

The probability of freedom from poliovirus infection in Australia, assuming surveillance practices meet the WHO target AFP case notification and stool sampling rates for each jurisdiction, is summarised in Table 4. As for Table 2, results were generated assuming a conservative initial prior probability of infection (0.5), and using the highest estimate of the probability of disease introduction (Beta(1,361)).

**Table 4:** Probability of freedom from poliovirus infection in Australia given continuous negative surveillance findings based on WHO-recommended notification and stool sampling practices

	Probability of freedom (%)^‡^			
**Design prevalence**	**10^-5^**		**10^-6^**	

**Time period**	**No age effect**	**Age effect**	**No age effect**	**Age effect**

6 months	83.0 (74.3-90.2)	94.0 (86.7-97.7)	53.4 (51.6-55.2)	56.3 (53.8-59.1)
1 year	95.4 (88.7-98.5)	99.2 (96.9-99.8)	56.7 (52.7-60.2)	62.3 (57.2-67.4)
2 years	99.1 (96.5-99.9)	99.7 (98.5-100.0)	62.8 (55.0-69.4)	72.6 (63.1-80.7)
3 years	99.4 (97.2-99.9)	99.7 (98.6-100.0)	68.4 (56.9-77.1)	80.5 (68.2-89.0)
4 years	99.4 (97.2-100.0)	99.7 (98.6-100.0)	73.2 (58.7-83.1)	86.0 (72.0-93.8)
5 years	99.4 (97.2-100.0)	99.7 (98.6-100.0)	77.3 (60.2-87.6)	89.7 (75.0-96.3)
10 years	99.4 (97.2-100.0)	99.7 (98.6-100.0)	88.7 (65.3-96.9)	95.4 (81.1-99.4)
20 years	99.4 (97.2-100.0)	99.7 (98.6-100.0)	92.7 (68.2-99.3)	96.0 (82.1-99.7)

^‡^Median (5^th^-95^th ^percentile), probability of introduction Beta(1,361)

If WHO targets were met for case notification and stool sampling rates, the median probability of Australia being free from poliovirus infection after 5 years of continuous negative surveillance results, assuming no difference in the risk of poliovirus infection with age, was 99.4% for a design prevalence of 10^-5^, and 77.3% for a design prevalence of 10^-6^. If the risk of infection was greater among younger children, the median probability of Australia being free from poliovirus infection after 5 years was 99.7% for a design prevalence of 10^-5^, and 89.7% for a design prevalence of 10^-6 ^(Table 4).

The estimated median sensitivity of AFP surveillance in Australia based on historical case notification and stool sampling rates, assuming no difference in the risk of infection with age and a design prevalence of 10^-5 ^(8.2%) was considerably lower than if WHO criteria were met for case notification and stool sampling rates (23.8%; sensitivity ratio 0.35). Assuming an increased risk of infection in younger children, the sensitivity of surveillance based on historical case notification and stool sampling rates (13.8%) was also considerably lower than if WHO criteria were met for case notification and stool sampling rates (37.6%; sensitivity ratio 0.37). Findings were similar when surveillance sensitivity was estimated using a design prevalence of 10^-6^, where sensitivity ratios assuming no difference in the risk of infection with age and an increased risk of infection in younger children both approximated 0.32.

The estimated sensitivity of AFP surveillance for each jurisdiction based on historical surveillance data is summarised in Table 3, along with sensitivity ratios comparing these estimates with those based on WHO recommended surveillance practices. The jurisdiction-specific sensitivity estimates in Table 3 are dependent on the size of the population under surveillance, and as such cannot be compared between jurisdictions; however, the sensitivity ratios can be compared. The jurisdictions that had the highest rate of stool sampling (Queensland and the Australian Capital Territory) had the highest sensitivity ratios.

### Maximum probability of disease freedom

For surveillance scenarios where the probability of disease introduction is lower than the surveillance system sensitivity in each surveillance period, the accumulation of continuous negative surveillance findings enables the surveillance system to establish a higher certainty of disease freedom than is possible based on the findings of any single surveillance period. In these scenarios, the maximum probability of disease freedom occurs when the value of additional surveillance information is not associated with an increased probability of disease freedom due to the risk of disease importation and temporal discounting of previous surveillance information.

For surveillance scenarios where the probability of disease introduction is lower than the surveillance system sensitivity, and these parameters can be considered constant over time, we found that the deterministic expected equilibrium posterior probability of disease freedom (PFreeEquil), which represents a maximum value in the scenarios examined here, can be estimated based on the mean probability of disease introduction (pIntro) and mean surveillance system sensitivity (SSe) using Equation 2. The expected maximum prior probability of freedom in these scenarios can also be estimated as pIntro/SSe. See additional file 1: PFreeEquil_proof for the derivation of this formula.(2)

Equation 2 can be used to understand the scenario tree model results, and explore the impact of alternative probabilities of disease introduction or surveillance sensitivity on the maximum (equilibrium) probability of disease freedom. Estimates of the expected maximum probability of disease freedom are derived only from parameter mean values, and differ slightly from the full simulation model results which are derived from probability distributions.

Based on mean estimates of the probability of introduction of 0.0028 (Beta(1,361)) and a mean surveillance system sensitivity of 0.084, the expected maximum probability of freedom is 96.9%. The expected maximum probability of freedom is achieved after approximately 10 years of surveillance given an initial prior probability of freedom of 0.5, with a probability of 96.0% achieved in just over 4 years. This expected maximum probability of freedom estimate is comparable to the simulation mean probability of freedom of 96.8% following 20 years of continuous negative surveillance findings. If the probability of introduction was assumed to be 0.0006 (Beta(2,3600)), the expected maximum probability of freedom of 99.4% (which equals the simulation mean probability of freedom following 20 years of surveillance) is achieved after approximately 9 years of surveillance, with a probability of 99.0% achieved in approximately 5 years.

If it is assumed that younger children are at increased risk of infection, and using a probability of introduction of 0.0028, the expected maximum posterior probability of freedom of 98.3% is only marginally higher than the model assuming no age effect, although this maximum probability is achieved in just over 5 years, with 98.0% achieved in approximately 3 years. If the probability of introduction was assumed to be 0.0006, the expected maximum probability of freedom is 99.7%, and is achieved after approximately 5 years of surveillance, with 99.0% reached within approximately 3 years.

## Discussion

As the world approaches polio eradication, the use of case-free periods as an indicator of the cessation of disease transmission becomes increasingly imprecise due to the high proportion of subclinical infections [46]. We found that the sensitivity of any single month of AFP surveillance in Australia based on historical data was low, although continuous negative surveillance over several years can produce a high level of accumulated surveillance sensitivity. Our findings emphasise, similar to previous modelling studies [31,46], that even after five years without a detected case, freedom from poliovirus infection cannot be assumed.

Sustained sensitive surveillance is required to demonstrate freedom from poliovirus infection in Australia and support the prevention of polio re-emergence. Based on historical surveillance practices, our findings suggest that establishing over 95% certainty of freedom from poliovirus infection at a design prevalence of 10^-5 ^requires continuous negative surveillance results for approximately four years assuming no differential risk of infection by age, or two years if it is assumed that there is an increased risk of infection in younger children.

Attainment of the target AFP notification rate of 1 case per 100,000 children aged less than 15 years is a critical component of the WHO assessment of surveillance adequacy. There is considerable variation in the rates of AFP reported internationally [47], and studies suggest that the true rate of AFP in Australia is not lower than the WHO target rate [41,42]. Using pooled data we found that the average AFP notification rate for most jurisdictions was not significantly different from the WHO target rate; however, stool sampling rates were generally significantly lower than the WHO recommended rate.

The ability of all States and Territories to meet the WHO surveillance targets, particularly with respect to stool sampling rates, would substantially improve the sensitivity of surveillance for poliovirus infection in Australia over short time periods. If the WHO surveillance targets were achieved in each jurisdiction, only approximately 2 years of negative surveillance findings would be required to establish 99% certainty of freedom from poliovirus infection at a prevalence of 10^-5 ^assuming no differential risk of infection by age, or 1 year of surveillance if it is assumed that there is an increased risk of infection among younger children.

The low rate of stool sampling in Australia compared with the WHO surveillance target may compromise the timeliness and effectiveness of the public health response in the event of poliovirus importation or re-emergence [48]. Despite the introduction of a series of strategies to facilitate the early notification and stool testing of paediatric AFP cases, including a large-scale campaign in 1998 [17], there has been little sustained improvement in notification rates. The stool sampling rate among eligible AFP cases reported in 2007 was the highest on record at 52% [22], although this remains well below the WHO target rate. It is clear that there are widespread and longstanding difficulties in adherence to the WHO recommended stool sampling procedures in Australia.

The long absence of polio in Australia, the failure of clinicians to associate AFP surveillance with non-polio AFP, and the presence of a confirmed alternative diagnosis have been identified as contributing to the under-reporting in AFP surveillance [17]. Anecdotal evidence suggests that clinicians are able to rationalise the low rate of stool sampling on clinical grounds. The unwillingness to obtain stool samples may be a product of the specialist management of cases in Australia, and the availability of sophisticated diagnostic technologies allowing rapid definitive diagnosis of cases with non-polio AFP. The WHO surveillance criteria have been considered less appropriate for the evaluation of AFP surveillance in developed communities with access to sophisticated diagnostic techniques [17].

More comprehensive stool sampling among AFP cases would increase the sensitivity of surveillance for poliovirus infection based on the WHO AFP-based surveillance model. However, the extent to which stool sampling rates reflect real deficiencies in the timeliness and sensitivity of AFP surveillance in the context of a population which generally has good access to a highly skilled specialist medical workforce remains to be established. Further work is needed to understand the clinical decision-making processes leading to the failure to notify or obtain stool samples from children presenting with AFP, and critically evaluate the basis of stool sampling for poliovirus surveillance in Australia.

Ensuring sustainable surveillance for polioviruses is a key challenge facing the Global Polio Eradication Initiative, as only 20 per cent of countries in polio-free regions are achieving WHO certification standard surveillance [49]. The sensitivity of AFP surveillance for poliovirus infection is limited by the high proportion of subclinical infections and low stool sampling rates. Alternative surveillance processes may be required to improve the sensitivity of surveillance in polio-free regions. Environmental surveillance for polioviruses can provide high sensitivity for poliovirus detection, and may be a useful supplementary surveillance strategy as global polio eradication approaches [50,51].

Well developed methods for the analysis of poliovirus surveillance information from multiple sources are also required to ensure that surveillance is adequate to detect infection at the desired population prevalence, and that resources for surveillance are used efficiently. Scenario tree models provide a transparent and flexible method for analysis that can integrate accumulated surveillance information from multiple surveillance system components [19]. As demonstrated by the present study, scenario tree modelling allows the systematic quantitative evaluation of the adequacy of surveillance systems, accounting for multiple factors that influence the probability of disease and the probability of detecting disease.

Uncertainty associated with the probability of introduction and establishment of poliovirus in Australia is a limitation of this analysis. The probability of poliovirus introduction and establishment is likely to fluctuate, and depend on trends in global surveillance and control; immunity among residents and travellers; and global mobility. In recognition of the significant risk of poliovirus importation, a World Health Assembly resolution on the immunisation of travellers from endemic areas has been proposed [4] to reduce the international spread of polioviruses.

Global eradication of polio remains achievable [7], and certification of the world as free of indigenous wild poliovirus is expected to occur three years after the last case of polio has been identified [52]. Although the risk of polio resurgence following certification is considered remote [52], the sensitivity of global surveillance is of critical importance in evaluating the likelihood of interrupting poliovirus transmission and disease freedom. Scenario tree modelling provides a valuable tool for systematically assessing the effectiveness of surveillance for poliovirus infection and identifying gaps in surveillance. The limitations of global surveillance, where they exist, need to be recognised and remedied to ensure progress towards disease eradication.

## Conclusion

Effective surveillance systems are critical for the global eradication of polio. Ensuring a high probability of freedom from poliovirus infection in Australia at a low level of disease prevalence requires sensitive long-term surveillance. The low rate of stool sampling among notified cases is an important modifiable factor contributing to the low sensitivity of AFP surveillance in Australia compared with WHO surveillance targets. Our findings emphasise the importance of maintaining sensitive surveillance beyond the period required for WHO certification, both to support global progress towards polio eradication, and to enable effective public health response in the event of the importation or re-emergence of polio.

## Competing interests

The authors declare that they have no competing interests.

## Authors' contributions

REW, PAJM, HK, and BM designed the study, REW and PAJM conducted the analysis, REW drafted the manuscript and REW, PAJM, HK, BM and CW performed critical revision of the manuscript content. All authors read and approved the final manuscript.

## Pre-publication history

The pre-publication history for this paper can be accessed here:

http://www.biomedcentral.com/1471-2334/9/162/prepub

## Supplementary Material

Additional file 1 **Proof of calculation of PFreeEquil**. Derivation of the formula for PFreeEquil. Click here for file

## References

[B1] Anon Global polio eradication initiative Bull World Health Organ 2006 84 8 595 PMC2627439 16917643

[B2] Global polio eradication initiative annual report 2007: Impact of the intensified eradication effort http://www.polioeradication.org/content/publications/AnnualReport2007_English.pdf

[B3] Progress toward interruption of wild poliovirus transmission--worldwide, January 2005-March 2006 MMWR Morb Mortal Wkly Rep 2006 55 16 458 462 16645572

[B4] World Health Organisation Conclusions and recommendations of the Advisory Committee on Poliomyelitis Eradication, November 2008 Wkly Epidemiol Rec 2009 84 3 17 28 19149015

[B5] Aylward RB Maher C Interrupting poliovirus transmission -- new solutions to an old problem Biologicals 2006 34 2 133 139 10.1016/j.biologicals.2006.02.012 16682220

[B6] World Health Organisation Polio Eradication Group Progress toward interruption of wild poliovirus transmission--worldwide, January 2005-March 2006 MMWR Morb Mortal Wkly Rep 2006 55 16 458 462 16645572

[B7] Ong BK Fisher DA Infectious disease eradication: poliomyelitis as a lesson in why "close" is not good enough Ann Acad Med Singapore 2005 34 10 593 594 16382242

[B8] Dowdle WR Birmingham ME The biologic principles of poliovirus eradication The Journal of Infectious Diseases 1997 175 Suppl 1 S286 292 9203732 10.1093/infdis/175.Supplement_1.S286 PMC7110371

[B9] Shibuya K Murray CJL Murray CL, Lopez AD, Mathers CD Poliomyelitis The global epidemiology of infectious diseases 2004 4 Geneva: World Health Organisation 111 149

[B10] Arita I Nakane M Fenner F Public health. Is polio eradication realistic? Science 2006 312 5775 852 854 10.1126/science.1124959 16690846

[B11] Major milestone reached in global polio eradication: Western Pacific Region is certified polio-free https://www.who.int/inf-pr-2000/en/pr2000-71.html

[B12] World Health Organisation Acute flaccid paralysis (AFP) surveillance: the surveillance strategy for poliomyelitis eradication Wkly Epidemiol Rec 1998 73 16 113 117

[B13] Stewardson AJ Roberts JA Beckett CL Prime HT Loh PS Thorley BR Daffy JR Imported case of poliomyelitis, Melbourne, Australia, 2007 Emerg Infect Dis 2009 15 1 63 65 10.3201/eid1501.080791 19116053 PMC2660702

[B14] Kennett ML Brussen KA Wood DJ Avoort HG van der Ras A Kelly HA Australia's last reported case of wild poliovirus infection Commun Dis Intell 1999 23 77 79 10323040 10.33321/cdi.1999.23.9

[B15] D'Souza RM Elliott E Polio eradication Commun Dis Intell 1999 23 3 76 10323039 10.33321/cdi.1999.23.8

[B16] Smith J Leke R Adams A Tangermann RH Certification of polio eradication: process and lessons learned Bull World Health Organ 2004 82 1 24 30 15106297 PMC2585869

[B17] D'Souza RM Kennett M Antony J Herceg A Harvey B Longbottom H Elliott E Surveillance of acute flaccid paralysis in Australia, 1995-97. Australian Paediatric Surveillance Unit J Paediatr Child Health 1999 35 6 536 540 10.1046/j.1440-1754.1999.00413.x 10634978

[B18] Whitfield K Kelly H Notification of patients with acute flaccid paralysis since certification of Australia as polio-free J Paediatr Child Health 2004 40 8 466 469 10.1111/j.1440-1754.2004.00429.x 15265189

[B19] Martin PAJ Cameron AR Greiner M Demonstrating freedom from disease using multiple complex data sources 1: A new methodology based on scenario trees Preventive Veterinary Medicine 2007 79 71 97 10.1016/j.prevetmed.2006.09.008 17224193

[B20] CDATA Online: 2006 Census (cat.no. 2064.0) http://www.abs.gov.au/CDATAOnline

[B21] Nathanson N Martin JR The epidemiology of poliomyelitis: enigmas surrounding its appearance, epidemicity, and disappearance Am J Epidemiol 1979 110 6 672 692 400274 10.1093/oxfordjournals.aje.a112848

[B22] Roberts JA Grant KA Ibrahim A Thorley BR Annual report of the Australian National Poliovirus Reference Laboratory, 2007 Commun Dis Intell 2008 32 3 308 315 19062766 10.33321/cdi.2008.32.31

[B23] Gidding HF Backhouse JL Gilbert GL Burgess MA National serosurvey of poliovirus immunity in Australia, 1996-99 Aust N Z J Public Health 2005 29 1 48 52 10.1111/j.1467-842X.2005.tb00748.x 15782872

[B24] Department of Health and Ageing Communicable diseases surveillance: Additional reports Commun Dis Intell 2003 27 3 419 427

[B25] Department of Health and Ageing Communicable diseases surveillance: Highlights for 2nd quarter, 2006 Commun Dis Intell 2006 30 3 385 405 17120495 10.33321/cdi.2006.30.38

[B26] McIntyre PB Heath TC O'Brien ED Hull BP National immunisation coverage--interpreting the first three quarterly reports from the ACIR Commun Dis Intell 1998 22 6 111 112 9648372 10.33321/cdi.1998.22.24

[B27] Fine PEM Ritchie S Perspective: Determinants of the severity of poliovirus outbreaks in the post eradication era Risk Analysis 2006 26 6 1533 1540 10.1111/j.1539-6924.2006.00855.x 17184395

[B28] Selvey C Immunisation coverage estimates Commun Dis Intell 2000 24 8 260 11022394 10.33321/cdi.2000.24.43

[B29] Centers for Disease Control and Prevention Resurgence of wild poliovirus type 1 transmission and consequences of importation--21 countries, 2002-2005 MMWR Morb Mortal Wkly Rep 2006 55 6 145 150 16484977

[B30] Cannon RM Demonstrating disease freedom-combining confidence levels Preventive Veterinary Medicine 2002 52 3-4 227 249 10.1016/S0167-5877(01)00262-8 11849719

[B31] Debanne SM Rowland DY Statistical certification of eradication of poliomyelitis in the Americas Math Biosci 1998 150 1 83 103 10.1016/S0025-5564(98)00007-8 9654894

[B32] Singh J Sharma RS Verghese T Epidemiological considerations on age distribution of paralytic poliomyelitis J Trop Pediatr 1996 42 4 237 241 10.1093/tropej/42.4.237 8816037

[B33] Horstmann DM Poliomyelitis: severity and type of disease in different age groups Ann N Y Acad Sci 1955 61 4 956 967 10.1111/j.1749-6632.1955.tb42554.x 13340604

[B34] Moore M Katona P Kaplan JE Schonberger LB Hatch MH Poliomyelitis in the United States, 1969-1981 J Infect Dis 1982 146 4 558 563 6288811 10.1093/infdis/146.4.558

[B35] Centers for Disease Control and Prevention Outbreak of polio in adults--Namibia, 2006 MMWR Morb Mortal Wkly Rep 2006 55 44 1198 1201 17093385

[B36] Prevots DR Ciofi degli Atti ML Sallabanda A Diamante E Aylward RB Kakariqqi E Fiore L Ylli A Avoort H van der Sutter RW Outbreak of paralytic poliomyelitis in Albania, 1996: high attack rate among adults and apparent interruption of transmission following nationwide mass vaccination Clin Infect Dis 1998 26 2 419 425 10.1086/516312 9502465

[B37] Bernier RH Some observations on poliomyelitis lameness surveys Rev Infect Dis 1984 6 Suppl 2 S371 375 6740075 10.1093/clinids/6.supplement_2.s371

[B38] Hinman AR Koplan JP Orenstein WA Brink EW Nkowane BM Live or inactivated poliomyelitis vaccine: an analysis of benefits and risks Am J Public Health 1988 78 3 291 295 10.2105/AJPH.78.3.291 3277452 PMC1349179

[B39] Tebbens RJ Pallansch MA Kew OM Caceres VM Jafari H Cochi SL Sutter RW Aylward RB Thompson KM Risks of paralytic disease due to wild or vaccine-derived poliovirus after eradication Risk Anal 2006 26 6 1471 1505 10.1111/j.1539-6924.2006.00827.x 17184393

[B40] Lam RM Tsang TH Chan KY Lau YL Lim WL Lam TH Leung NK Surveillance of acute flaccid paralysis in Hong Kong: 1997 to 2002 Hong Kong Med J 2005 11 3 164 173 15951581

[B41] Morris AM Elliott EJ D'Souza RM Antony J Kennett M Longbottom H Acute flaccid paralysis in Australian children J Paediatr Child Health 2003 39 1 22 26 10.1046/j.1440-1754.2003.00065.x 12542807

[B42] Whitfield K Kelly H Using the two-source capture-recapture method to estimate the incidence of acute flaccid paralysis in Victoria, Australia Bull World Health Organ 2002 80 11 846 851 12481205 PMC2567677

[B43] Grassly NC Fraser C Wenger J Deshpande JM Sutter RW Heymann DL Aylward RB New strategies for the elimination of polio from India Science 2006 314 5802 1150 1153 10.1126/science.1130388 17110580

[B44] Vose D Risk Analysis: A quantitative guide 2000 2 Chichester: John Wiley & Sons

[B45] Chen X Zhou K Aravena JL On the binomial confidence interval and probabilistic robust control Automatica 2004 40 1787 1789 10.1016/j.automatica.2004.04.016

[B46] Eichner M Dietz K Eradication of poliomyelitis: when can one be sure that polio virus transmission has been terminated? Am J Epidemiol 1996 143 8 816 822 8610692 10.1093/oxfordjournals.aje.a008820

[B47] Harris BN Durrheim DN Ogunbanjo GA Polio eradication--the validity of surveillance indicators Trop Med Int Health 2003 8 5 386 391 10.1046/j.1365-3156.2003.01048.x 12753631

[B48] Durrheim DN Massey IP Kelly H Re-emerging poliomyelitis--is Australia's surveillance adequate? Commun Dis Intell 2006 30 3 275 277 17120482 10.33321/cdi.2006.30.25

[B49] Global Polio Eradication Initiative Strategic Plan 2009-2013 Framework Document http://www.polioeradication.org/content/publications/PolioStrategicPlan09-13_Framework.pdf

[B50] Deshpande JM Shetty SJ Siddiqui ZA Environmental surveillance system to track wild poliovirus transmission Appl Environ Microbiol 2003 69 5 2919 2927 10.1128/AEM.69.5.2919-2927.2003 12732567 PMC154486

[B51] Huang QS Greening G Baker MG Grimwood K Hewitt J Hulston D van Duin L Fitzsimons A Garrett N Graham D Persistence of oral polio vaccine virus after its removal from the immunisation schedule in New Zealand Lancet 2005 366 9483 394 396 10.1016/S0140-6736(05)66386-6 16054940

[B52] Dutta A Epidemiology of poliomyelitis--options and update Vaccine 2008 26 45 5767 5773 10.1016/j.vaccine.2008.07.101 18755232

