# Supplementary material for: An evaluation of the sensitivity of acute flaccid paralysis surveillance for poliovirus infection in Australia
Source: BMC Infect Dis. 2009 Sep 30;9:162. doi: 10.1186/1471-2334-9-162 (PMC2761398; doi:10.1186/1471-2334-9-162)
Supplement: Additional file 1 — Proof of calculation of PFreeEquil. Derivation of the formula for PFreeEquil. [file 1471-2334-9-162-S1.PDF]

Proof of calculation of PFreeEquil

This document contains additional material to accompany the paper, "An evaluation of the sensitivity of acute flaccid paralysis surveillance for poliovirus infection in Australia" that was provided by Dr Evan Sergeant, Director, AusVet Animal Health Services, Orange NSW 2800, Australia. evan@ausvet.com.au

Terminology

PPInf	= Prior probability of infection
PPFree	= Prior probability of freedom
PFree	= Posterior probability of freedom
PFreeEquil	= Stable posterior probability of freedom
PIntro	= Probability of introduction
SSe	= System sensitivity

Assumptions

- SSe and PIntro remain constant
- SSe > PIntro
- PFree reaches a constant level in the long term (PFreeEquil) where the effects of ongoing surveillance and probability of introduction are balanced and PFree therefore remains constant until either PIntro or SSe change. PFreeEquil may be a stable maximum or minimum, depending on the balance between the opposing effects of SSe and PIntro, and the initial PPInf nominated.
- Note, where SSe = PIntro, PFreeEquil = 0

Calculating the prior probability of freedom (PPFree)

For a stable posterior probability of freedom, PFree remains constant at each time point, as do PPInf and consequently PPFree. As such we can remove time from the calculation of PPInf [1] (p. 95), and the equation for calculating PPInf becomes:

$$\text{PPInf} = 1 - \text{PFreeEquil} + \text{PIntro} - \text{Pintro}\,(1 - \text{PFreeEquil})$$

This simplifies to: $$\text{PPInf} = 1 - \text{PFreeEquil} + \text{PIntro.PFreeEquil}$$

Or: $$\text{PPFree} = 1 - (1 - \text{PFreeEquil} + \text{PIntro. PFreeEquil})$$

Which simplifies to: $$\text{PPFree} = \text{PFreeEquil}\,(1 - \text{PIntro}) \qquad \text{(A)}$$

Calculating the equilibrium posterior probability of freedom (PFreeEquil)

From [1] p.94: $$\text{PFreeEquil} = (1 - \text{PPInf}) / (1 - \text{PPInf.SSe})$$

Substituting PPFree: $$\text{PFreeEquil} = \text{PPFree} / (1 - (1 - \text{PPFree})\,\text{SSe})$$

Substituting (A) above: $$\text{PFreeEquil} = \text{PFreeEquil}\,(1 - \text{PIntro}) / (1 - (1 - \text{PFreeEquil}\,(1 - \text{PIntro}))\,\text{SSe})$$

Cancelling PFreeEquil: $$1 = (1 - \text{PIntro}) / (1 - (1 - \text{PFreeEquil}\,(1 - \text{PIntro}))\,\text{SSe})$$

Or: $$1 - \text{PIntro} = 1 - (1 - \text{PFreeEquil}\,(1 - \text{PIntro}))\,\text{SSe}$$

And: $$\text{PIntro} = (1 - \text{PFreeEquil}\,(1 - \text{PIntro}))\,\text{SSe}$$

Rearranging: $$\text{PIntro} / \text{SSe} = 1 - \text{PFreeEquil}\,(1 - \text{PIntro})$$

And: $$\text{PFreeEquil}\,(1 - \text{PIntro}) = 1 - (\text{PIntro} / \text{SSe})$$

Finally: $$\text{PFreeEquil} = (1 - (\text{PIntro} / \text{SSe})) / (1 - \text{PIntro})$$

Reference

1. Martin PAJ, Cameron AR, Greiner M: **Demonstrating freedom from disease using multiple complex data sources 1: A new methodology based on scenario trees**. *Preventive Veterinary Medicine* 2007, **79**:71-97.
